# Transcriptomic signature reveals mechanism of flower bud distortion in witches’-broom disease of soybean (*Glycine max*)

**DOI:** 10.1186/s12870-018-1601-1

**Published:** 2019-01-15

**Authors:** Sarika Jaiswal, Pravin V. Jadhav, Rahul Singh Jasrotia, Prashant B. Kale, Snehal K. Kad, Mangesh P. Moharil, Mahendra S. Dudhare, Jashminkumar Kheni, Amit G. Deshmukh, Shyamsundar S. Mane, Ravindra S. Nandanwar, Suprasanna Penna, Joy G. Manjaya, Mir Asif Iquebal, Rukam Singh Tomar, Prashant G. Kawar, Anil Rai, Dinesh Kumar

**Affiliations:** 1 Centre for Agricultural Bioinformatics, ICAR-Indian Agricultural Statistics Research Institute, Library Avenue, PUSA, New Delhi, 110012 India; 2 Post Graduate Institute, Dr. Panjabrao Deshmukh Krishi Vidyapeeth, Akola, Maharashtra, 444104 India; 3 National Research Centre on Plant Biotechnology, LBS Centre, PUSA Campus, New Delhi, 110012 India; 4 Department of Biotechnology, Junagadh Agricultural University, Junagadh, Gujarat India; 5 Nuclear Agriculture and Biotechnology Division, Homi Bhabha National Institute, Bhabha Atomic Research Centre (BARC), Trombay, Mumbai, 400 085 India; 6 ICAR- Directorate of Floricultural Research, College of Agriculture, Pune, Maharashtra, 411 005, India

**Keywords:** Soybean, Witches’ broom, Transcriptome, Assembly, Differential expression

## Abstract

**Background:**

Soybean (*Glycine max* L. Merril) crop is major source of edible oil and protein for human and animals besides its various industrial uses including biofuels. Phytoplasma induced floral bud distortion syndrome (FBD), also known as witches’ broom syndrome (WBS) has been one of the major biotic stresses adversely affecting its productivity. Transcriptomic approach can be used for knowledge discovery of this disease manifestation by morpho-physiological key pathways.

**Results:**

We report transcriptomic study using Illumina HiSeq NGS data of FBD in soybean, revealing 17,454 differentially expressed genes, 5561 transcription factors, 139 pathways and 176,029 genic region putative markers single sequence repeats, single nucleotide polymorphism and Insertion Deletion. Roles of PmbA, Zn-dependent protease, SAP family and auxin responsive system are described revealing mechanism of flower bud distortion having abnormalities in pollen, stigma development. Validation of 10 randomly selected genes was done by qPCR. Our findings describe the basic mechanism of FBD disease, right from sensing of phytoplasma infection by host plant triggering molecular signalling leading to mobilization of carbohydrate and protein, phyllody, abnormal pollen development, improved colonization of insect in host plants to spread the disease. Study reveals how phytoplasma hijacks metabolic machinery of soybean manifesting FBD.

**Conclusions:**

This is the first report of transcriptomic signature of FBD or WBS disease of soybean revealing morphological and metabolic changes which attracts insect for spread of disease. All the genic region putative markers may be used as genomic resource for variety improvement and new agro-chemical development for disease control to enhance soybean productivity.

**Electronic supplementary material:**

The online version of this article (10.1186/s12870-018-1601-1) contains supplementary material, which is available to authorized users.

## Background

Soybean (*Glycine max* L. Merril) caters the global need of edible oil (25%) and protein concentrate (67%) in animal feeds for livestock, poultry and fish. It contributes in meat, milk, cheese, bread, and oil. Per unit area, protein produced by this crop is highest, thus this crop has various epithets like “Cow of the field” or “Gold from soil”, “yellow jewel”, “great treasure”, “nature’s miracle protein” [1]. The top five global soybean producers which contribute more than 92% are, USA, Brazil, Argentina, China and India [2]. It is also grown in four major continents covering more than 20 countries including India. Soybean is a promising crop due to its potential beneficial roles in lowering of cholesterol, anti-carcinogenic effects, and protective effects against obesity, diabetes, irritants of the digestive tract, bone, and kidney diseases [3]. Soybean is also much more relevant as bioenergy crop for biofuel production along with their co-products as livestock feed [4]. Productivity of soybean is adversely affected by various biotic and abiotic stresses like salinity, pathogens, heat, drought, heat, soil heavy metals and compaction [5]. Among the biotic stresses, phytoplasma and viruses are major impediments causing productivity loss from 2 to 90% [6]. According to the earlier reports, several biotic factors have been identified in the host leading to reduced pollen vigour [7], seed mass variants [8], floral abnormalities, sepal hypertrophy, virescence, phyllody, aborted reproductive organs [9], enhanced vegetative growth [10], premature flower abortion [11], etc. Cytological examination of plant reproductive organs revealed distorted pollen grains in the symptomatic plants hampering pollination and fertilization [12].

Symptom and transmission of witches’ broom disease of soybean is well documented in India [13]. This disorder leads to failure of pod formation and lack the senescence maturity till the end of season. The symptoms are also not predictable. Abnormal development in the floral bud formation in witches’-Broom Disease of soybean causes, even acute loss in productivity [12]. It is referred in India as ‘floral bud distortion’ caused by phytoplasma [14, 15]. In USA and Iran, it is called bud proliferation syndrome [16], and pod set failure [17, 18], respectively.

Earlier gene expression studies on floral tissues are confines to microarray based limited differential gene expression [19]. Though RNA-Seq atlas of soybean has 14 diversified tissues including flower but no expression data is available for any abiotic and biotic stresses [20, 21]. Cytological and limited molecular study on FBD has been carried out revealing 27 DEG having ARF9 transcriptional factor and a regulatory protein FHA along with their protein-protein interaction network [12]. Though whole genome sequence of soybean has been done in 2010 [22] but without RNA-Seq approach detailed mechanism of FBD cannot be deciphered. Such knowledge discovery is not only required to understand basic mechanism of disease but is also relevant in designing of disease combating strategies in the endeavour of soybean productivity. By RNA-Seq approach stress responsive major morphological and physiological pathways in a single crop genotype has been successfully deciphered in crop like tea [23], tomato [24] and cassava [25]. In case of agricultural crop like soybean, stress response has been found specific to varieties/ accessions [26].

Present work aims to decipher key candidate genes and molecular mechanism in manifestation of flower bud distortion in witches’ Broom Disease of soybean. This study also aims to describe biochemical pathways along with genic region marker discovery (SSR, SNP and InDels) and prediction of TFs associate with FBD.

## Results and discussion

### Identification of symptomatic and asymptomatic plant of FBD

**Fig. 1:**
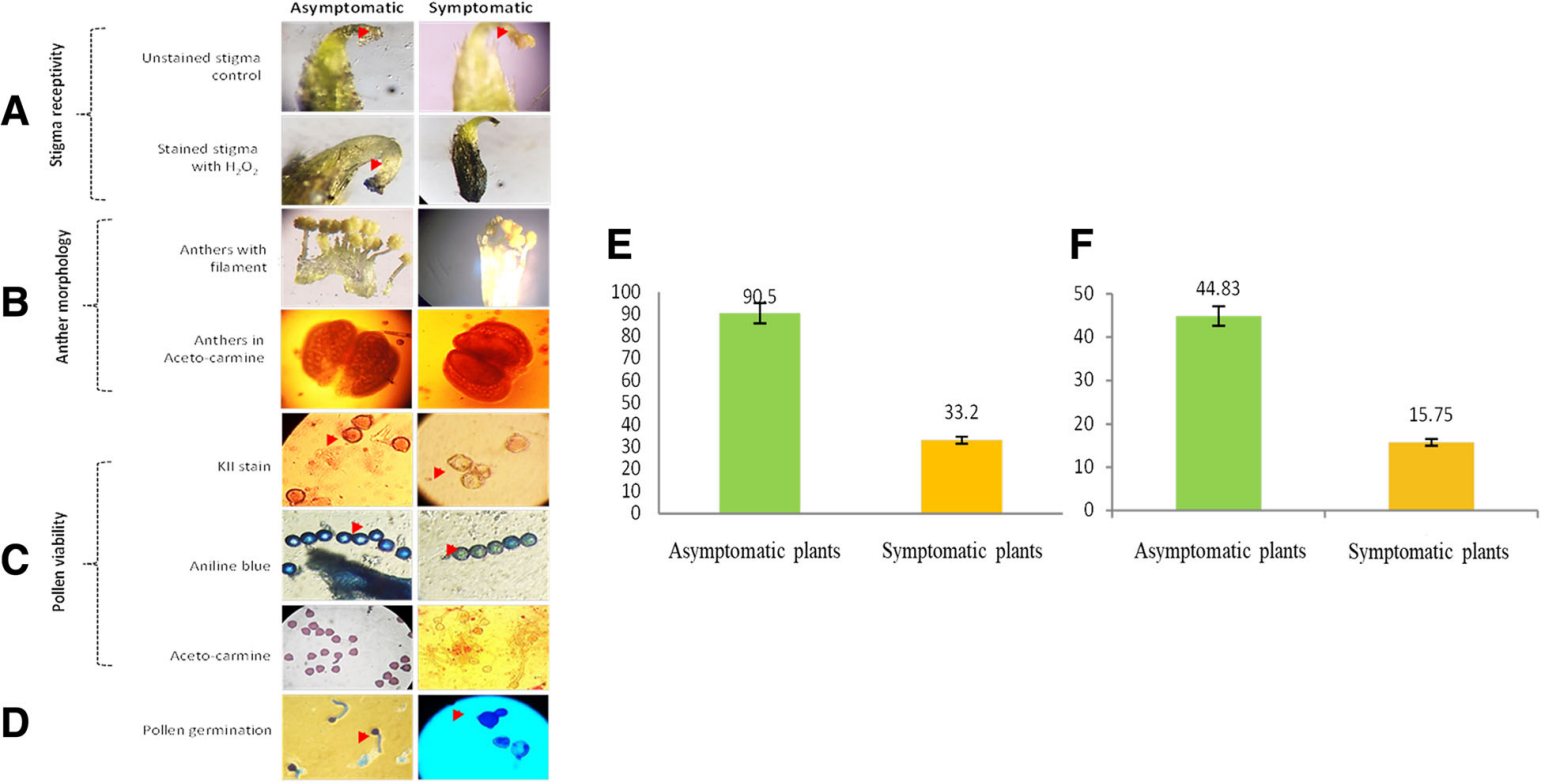
Cytological studies of floral reproductive organs in FBD symptomatic and asymptomatic soybean plant. **a** Stigma receptivity, **b** Anther morphology, **c** Percent pollen viability, **d** Pollen germination, **e** Percent pollen viability in FBD found reduced than asymptomatic, **f** Pollen germination ability in FBD found reduced than asymptomatic

**Fig. 2:**
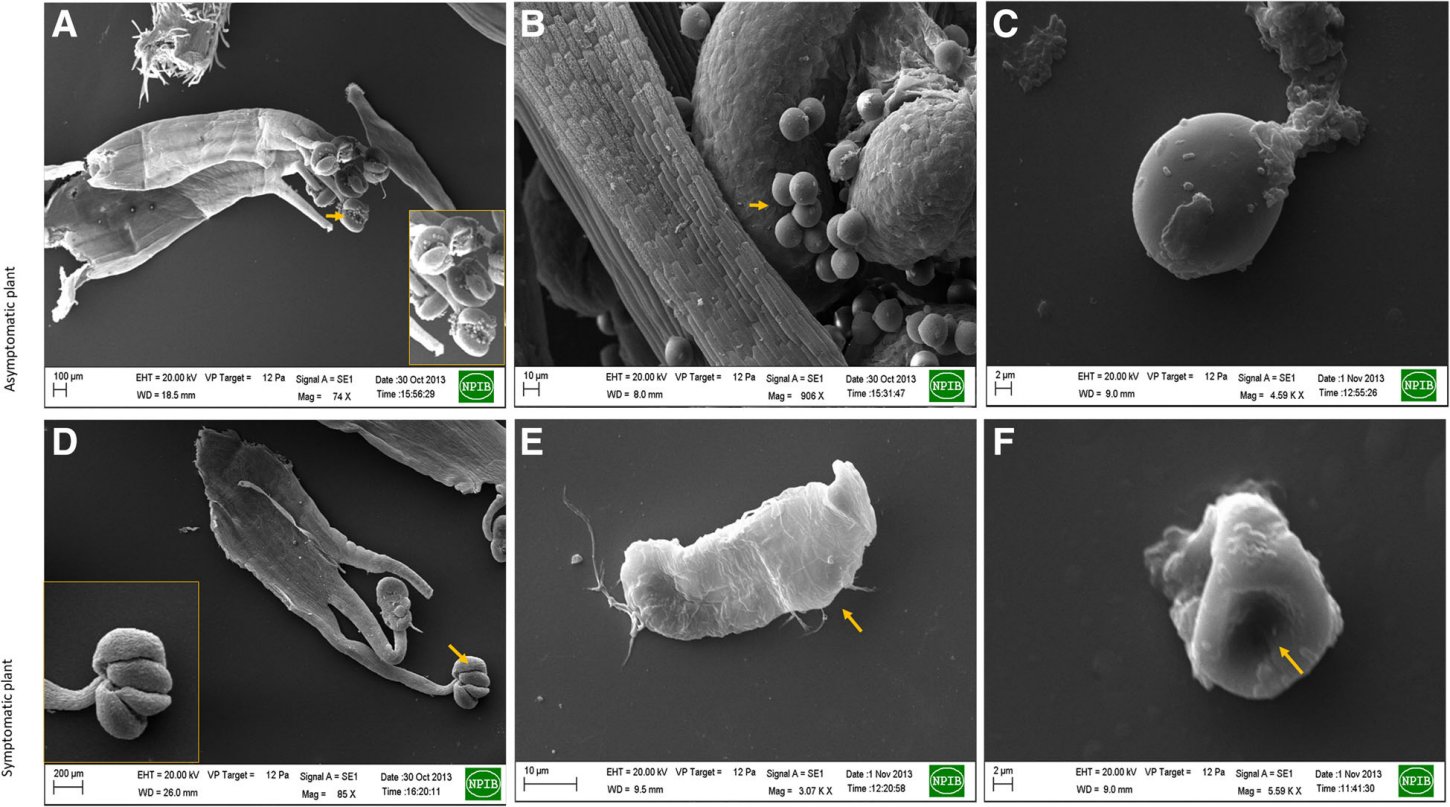
Scanning electron micrographs of floral organs of symptomatic and asymptomatic soybean plant **a** & **b**: Pollen on anthers of asymptomatic plant; **c** Pollen grain in asymptomatic plant; **d** Anthers with no/less number of pollens in symptomatic plant; E&F: Distorted pollen grains in the symptomatic plant

**Fig. 3:**
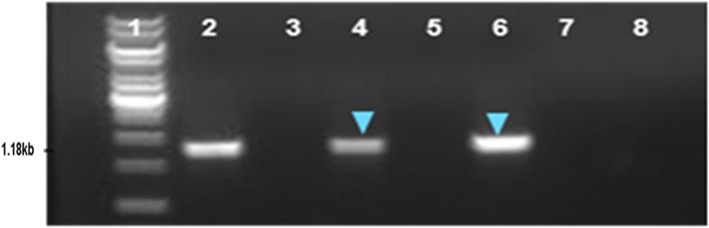
PCR based screening of symptomatic and asymptomatic soybean plants for phytoplasma using P1-P7/P1-P3 primers. Lane 1: Ladder; 2: Positive control; 3, 5 and 7: asymptomatic soybean plants; 4 and 6: symptomatic soybean plants; 8: Negative control

Discrimination of symptomatic and asymptomatic plants was done successfully by cytological studies of floral reproductive organs by pollen viability, morphology, stigma receptivity, anther morphology and germination assay (Fig. 1). SEM of floral organ, pollen and anther were also obtained to confirm the same (Fig. 2). Subsequently, they were further successfully confirmed by PCR based test by amplicon size as well as positive and negative control (Fig. 3). Since mycoplasma infection in asymptomatic plant is not easy to rule out, hence PCR based diagnostics is necessary [27].

### RNA-Seq data generation

A total of 30,669,583 and 36,967,121 paired end reads for both the sets, i.e., infected and control, respectively were generated. A total of 156,054 and 198,931 low quality reads from both the samples, were removed and remaining high quality reads were used for transcriptome analysis. Trinity assembler generated de novo assembly having 258,427 transcripts with default *k-*mer value 25 (Table 1). This was followed by removal of redundant sequences using CAP3 assembler, a total of 211,343 transcripts were obtained which were used for further analysis. Final assembly had GC content 41.96% with N50 of 1081 bp. Minimum and maximum transcript lengths were 190 bp and 50,081 bp, respectively.

**Table 1:** Reads’ statistics before and after trimming

Sample name	Raw paired-end reads pairs	Both Surviving	Forward Only Surviving	Reverse Only Surviving	Dropped
Infected	30,669,583	27,345,451 (89.16%)	2,814,040 (9.18%)	354,038 (1.15%)	156,054 (0.51%)
Control	36,967,121	32,541,568 (88.03%)	3,787,558 (10.25%)	439,064 (1.19%)	198,931 (0.53)

Though soybean genome is having 66,210 predicted gene model [20] but constructed transcriptome assembly appears to be represented by relatively higher number of transcripts (211343). Potential reasons for relatively higher number of observed transcripts could be (1) paleopolyploid origin of soybean genome having twice duplication event (59 MYA and 13 MYA), where variant transcripts are expected due to differential half-life/ retention time of homologues across genome having variation among them. These variations are due to recombination, structural variation due to insertion, deletion, inversion, unequal crossing-over, pseudogenization and background mutation [22, 28], (2) stress induced isoforms due to alternative splicing of immune associated genes [29] and (3) higher number of genes (> 66 K) and average number of exons per gene which varies from 3 to 5 is also expected to generate higher number of transcripts (196 to 330 thousand) [20].

### Identification of differentially expressed genes

Analysis of DEG were carried out by transcriptome assembly based approach because it is expected to discover “extra genes” [30] and isoforms [31] also retaining phytoplasma transcripts. However, mapping of transcripts over soybean reference genome assembly was done to evaluate the obtained DEG. Comparison of three different thresholds of mapped reads at 75, 90, 95 of % similarity was observed with < 5% variation (Table 2). This indicates accuracy of transcriptome assembly as well as uniformity of coverage and depth as there is < 5% variation.

**Table 2:** Mapping of transcripts over soybean reference genome assembly

Total number of transcripts	Total number of mapped transcripts over reference genome	Percentage mapped transcripts	Percentage similarity threshold
211,343	191,864	90.78	75
211,343	189,997	89.90	90
211,343	183,538	86.84	95

By transcriptome assembly approach, we found 35,725, 14,487 and 4490 DEGs at three different q value parameters i.e. q = 0.9, q = 0.95 and q = 0.99, respectively. DEGs were discovered by both the methods, i.e., edgeR and NOISeq. DEG discovered by NOISeq was compared with edgeR. Out of 17,454 DEGs obtained by edgeR, a total of 12,182 and 12,053 were found to be common in both at q = 0.9 and 0.95, respectively. At q = 0.99, comparison of both the methods showed 3879 common DEGs. Our comparative analyses by NOISeq validated that DEG obtained. Its comparison with edgeR at different Q values is presented in Table 3. At much higher stringency (q = 0.99), genes having higher expression (> ± 8 fold change value) more than 85% of the DEGs were found common. Graphical representation of upregulated and downregulated genes of these sets are depicted in heatmap (Additional file 1).

**Table 3:** Comparison of DEGs obtained by NOISeq and edgeR

Q values	NOISeq DEGs	Common DEGs in NOISeq and edgeR
q = 0.9	35,725	12,182
q = 0.95	14,487	12,053
q = 0.99	4490	3879

### Annotation and functional characterization

**Fig. 4:**
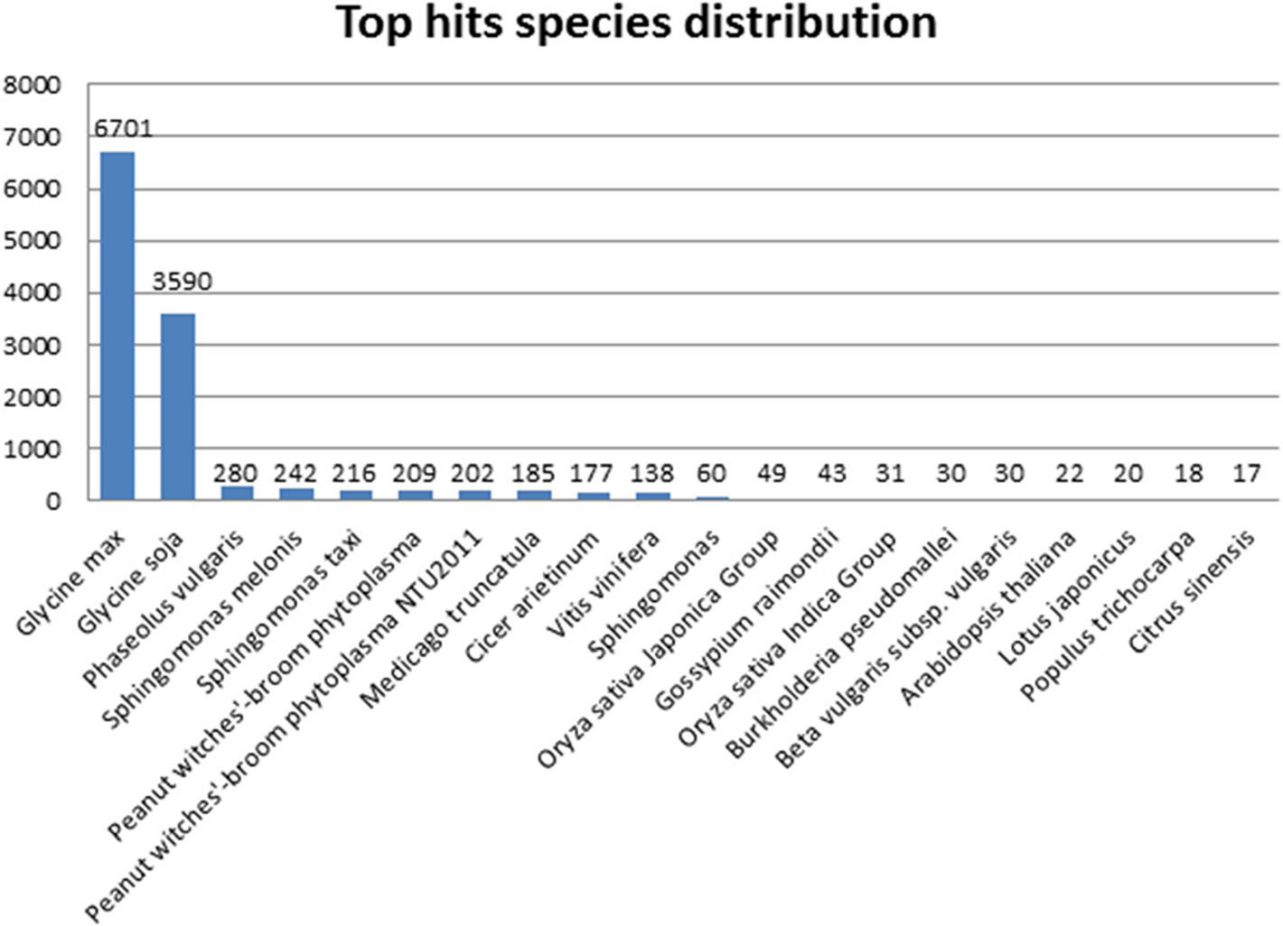
Top 20 species distribution of differential expressed genes of soybean

**Fig. 5:**
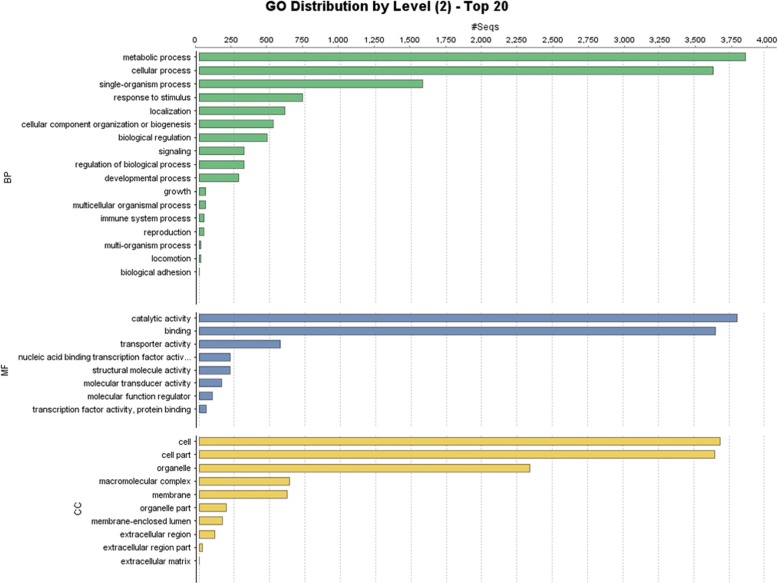
Gene ontology of differential expressed genes of soybean, Green color lines represented biological process, while blue and yellow color lines showed molecular functions and cellular components

Homology search of soybean differential expressed genes revealed a total of 12,900, out of 17,454 unigenes having similarity with other known gene in the database. Maximum number (6701) of hits matched with *Glycine max*, followed by *Glycine soja* and *Phaseolus vulgaris* i.e. 3590 and 280, respectively (Fig. 4, Additional file 2). There were only 411 phytoplasma specific transcripts, which is 2.35% of the total DEGs. Gene ontology analysis was performed to categorized genes into three sub-division of molecular, biological and cellular functions (Fig. 5).

From the total 17,454 differential expressed genes, 5561 unigenes showed similarity with transcriptional factors using blast against PlantTFDB v4.0. Maximum number of unigenes showed similarity with MYB i.e. 449, followed by bHLH, ERF, NAC and FAR1 in 439, 363, 339 and 296, respectively (Additional file 3). KEGG pathway analysis showed that 6041 unigenes were involved in 139 pathways (Additional file 4).

Among the DEGs hits of the flower bud tissue transcriptomic data of soybean, 296 hits were found with phytoplasma species. FBD transcriptome is mixture of transcripts of host and parasite both. This is due intra cellular presence of phytoplasma in symptomatic soybean samples. Many of the transcripts of host plants are in response to the requirement of phytoplasma. Phytoplasma is dependent on protein synthetic machinery of hosts for survival [32] as it contains very limited set of genes due to its smaller genome size (500-1500Kbp) [33]. Similar higher abundance of phytoplasma transcripts are already reported in other crops witches’ broom disease also like Paulownia [34]. Phytoplasma lowers the host plant systemic resistance by supressing salicylic acid pathways, enhancing its growth for its further invasion, proliferation and dispersion [35].

Among the DEGs we found up regulation of gene PmbA which is modulator of DNA gyrase. This is required for faster bacterial replication fork bidirectional DNA unwinding along with helicase [36]. Multiple sets of transcripts with hit as “retron-type reverse transcriptase” was found which is obviously expected in phytoplasma due to its role in genome size plasticity [37]. Its coding gene known as intron II reverse transcriptase is scattered over phytoplasma genome for lateral transfer of the genes as an adoptive mechanism of parasite to survive by creating variability. Different strains of same phytoplasma species varies in their size due to such genes [37].

We found differential expression of Zn-dependent protease transcripts which is known for virulence triggering gene in crop apple. The polymorphism of this gene has been found associated with moderate to severe disease condition. This Zn-dependent protease gene is also known as hflB (synonym ftsH) which codes for membrane associated ATP- and Zn2 + −dependent proteases controlling assembly, degradation and stability of protein affecting membrane and cytoplasm [38].

Up regulated virulence protein SAP54 which is also a type of effector, it mediates degradation of MTFs (MADS-box transcription factors) through 26S proteasome shuttle protein RAD23 resulting into floral bud distortion (FBD) along with development of phyllody (leafy flower). This phyllody is and adaptive response of phytoplasma by extending the vegetative tissue (leaf like flowers) to attract insect vector. This further enhances the insect colonization which is a strategic adaptation by phytoplasma for wider dispersal through insect vector [39].

Various transcripts of auxin responsive factor, auxin transport protein, auxin induced protein were found differentially expressed. They are known to play role in pollen wall-patterning and pollen development [40]. Auxin-responsive promoter elements are known to be involved in switching of vegetative to reproductive phase along with environmental signalling. Phytoplasma perturbs this normal reproductive development of floral tissue leading to FBD [12].

In witches’ broom syndrome (WBS), there is increase of carbohydrate protein and chlorophyll by > 2 fold [41]. This is due to impaired hormonal balance leading to impaired amino acid and carbohydrate translocation along with senescence. The vegetative period prolongs as phyllody but there is no proper development of pod.

Such flower virulence and distortion with abnormal shoot branching and stunted growth is also reported in brassica [42]. Sugar transport and accumulation leads to distant signalling for manifestation of FBD [9].

Upregulation was observed in SAP11 gene which encodes for phytoplasma protein effector is known to enhance insect vector reproduction by attenuating plant development and immunity [43]. Phytoplasma effectors are reported to alter development of flower, thus induces witches’ broom and also modify shape of the leaf to facilitate plant-insect interactions. These effectors facilitates advantage in lifecycle of phytoplasma into two different kingdoms namely, Plantae (soybean) and Animalia (insect) [44]. These effectors can be used in further research for development of new agrochemicals required in disease combating strategies [45].

### Mining of putative molecular markers

A total of 27,925 SSR markers were mined from 211,344 transcripts obtained by de novo transcriptome assembly of *Glycine max*. Table 4 provides the information of all repeat units found in mononucleotides, dinucleotides, trinucleotides, tetranucleotides, pentanucleotides and hexanucleotides markers. There is no polymorphism discovery as these SSRs are mined from a single genotype, however, the information generated (Additional file 5) having details of loci along with their computed primer for genotyping. Future use of these genomic resources were evaluated by in silico genotyping of SSR loci by e-PCR. For this, mined SSR loci having di and tri repeats were used for e-PCR validation. A total of 1874 transcripts were (size of > 1000 bps) were used for e-PCR. After exclusion of “non-specific” e-PCR products (having more than one hit with 100% similarity), a total of 193 specific SSR loci were found which can be used for future genotyping (Additional file 6). Non-specific e-PCR products represented by multiple hits were expected in soybean genome as it contains > 75% duplicate genes due to duplication events [22] and also due to isoforms. Our e-PCR results clearly demonstrates that for paleodiplod genome species like soybean, such approach can be successfully used before wet-lab validation of SSR loci to reduce the time and cost by exclusion of the multiple copy transcripts in genotyping. These final set of SSR loci can be used for genotyping required in diversity, QTL and association mapping.

**Table 4:** Information of mined simple sequence repeats and all repeating units

	De novo Transcriptome assembly
Sequences examined	211,344
Identified SSRs	29,964
SSR containing sequences	24,856
Sequences containing more than 1 SSR	4111
SSRs present in compound formation	2039
Mono	15,068
Di	7131
Tri	7348
Tetra	284
Penta	59
Hexa	74

**Fig. 6:**
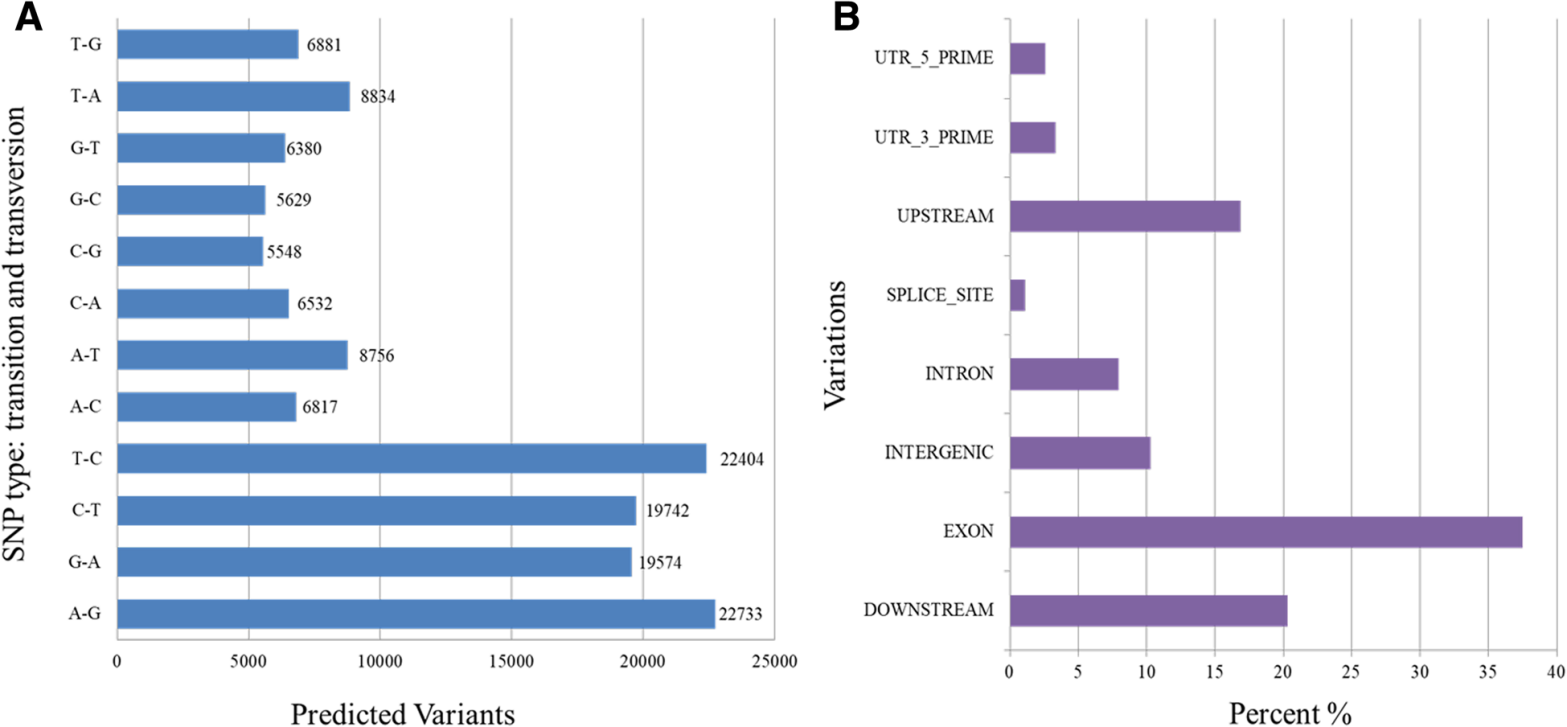
**a** Distribution of SNPs (Ts/Tv) in Soybean sequences; **b** Annotation and classification of Variants on the basis of locations

Though the transcriptomic data belongs to single genotype, JS- 335 Indian soybean variety but mining of SNP and InDels has been done successfully by aligning it over available reference genome which represents Williams 82 variety of USA. A total of 146,065 variants were found in soybean RNA-seq which contains 139,461 SNPs and 6604 InDels. Maximum variants were found in chromosome 18 i.e. 9582, followed by 8748 and 7489 in chromosome 6 and 2, respectively. Minimum number of variants were found in chromosome 11 i.e. 4739. The Ts/Tv rations of SNP was 1.53 and A-G and T-C were maximum predicted variants in transition whereas T-A and A-T were maximum predicted variants in transversion (Fig. 6a). Also, the annotation was performed against *Glycine max* genome where maximum variants (37.48%) were found in exonic region, followed by downstream (20.32%) and upstream (16.88%) regions, respectively (Fig. 6b; Additional file 7).

All the 176,029 putative markers (SSRs, SNPs and InDels) can be used as a genomic resource for future research especially for QTL, gene mapping and linkage analysis. Such use of DNA markers for phytoplasma resistance QTL mapping are already reported in crop like bitter-berry (*Prunus virginiana*) [46]. Phytoplasma resistance trait introgression in varietal improvement program using DNA markers has been successfully reported in apple [47]. In coconut, lethal yellowing disease (LYD) which is caused by phytoplasma DNA markers have been used in hybrid variety development program for improvement of LYD resistance [48].

### Validation and expression analysis by qPCR

**Fig. 7:**
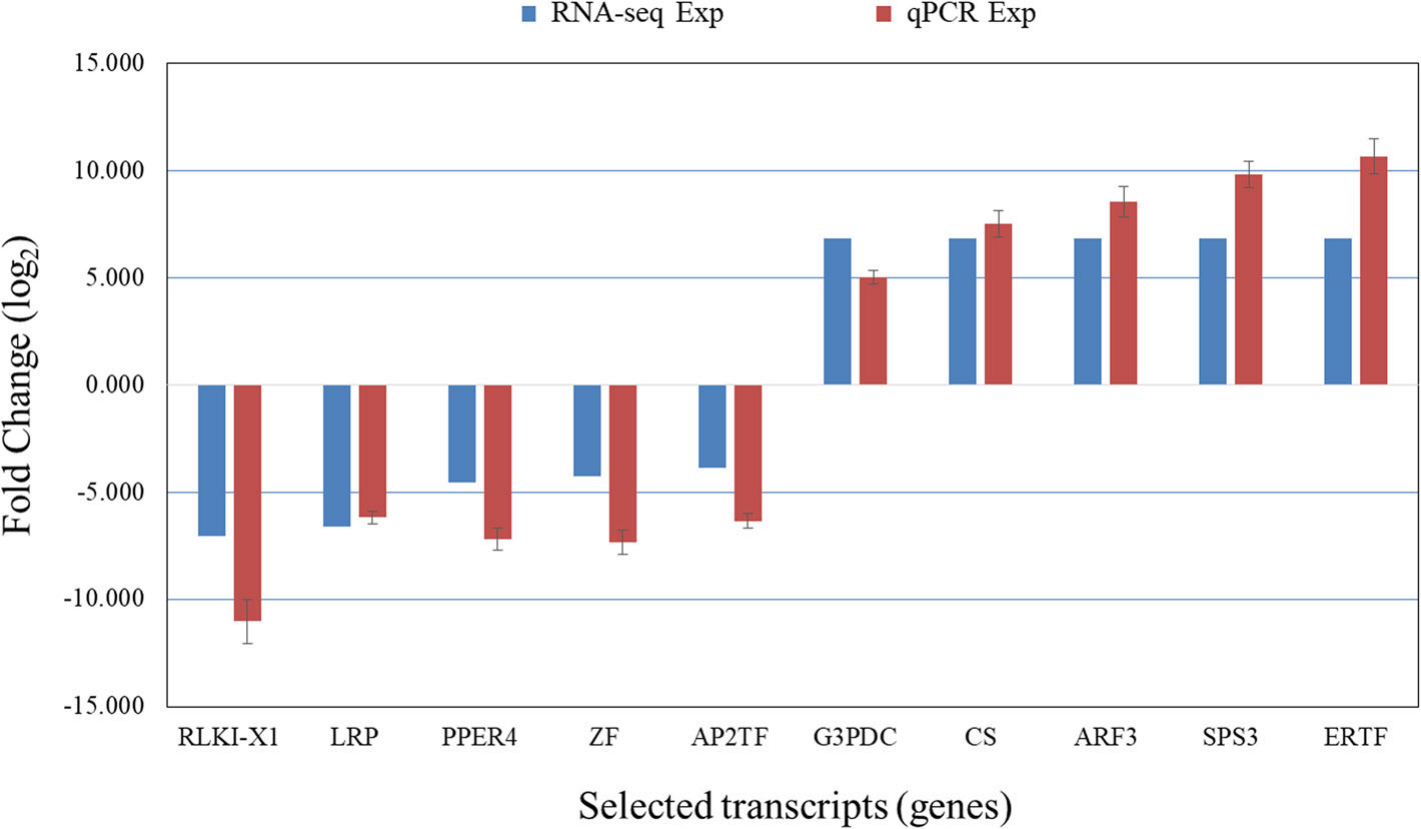
Comparison of gene expression between RNA-seq and qPCR for selected transcripts (genes)

Relative gene expression values obtained by qPCR analysis of all the 10 genes having up and down regulated, were in correspondence with computed log fold change value of DEGs (Fig. 7). Details of Gene and primers used for qPCR are listed in Table 5.

**Table 5:** Gene and primes sequence details of qPCR

Gene Name	Gene ID	Forward primer	Tm	Reverse primer	Tm
Receptor like kinase isoform X1	RLKI-X1	ACCAAACTCGGAACTTGATGGAGC	63.37	GTAGGCCTAAGTGTTGGAGAAGCA	62.37
Transcript ID: TRINITY_DN54263_c0_g1_i14					
La-related protein	LRP	GGCCCATTCCATTTCCAGCACG	64.61	TGCACATGAGGAAGAAGATGGGGA	63.71
Transcript ID: TRINITY_DN56761_c0_g1_i4					
Probable peroxygenase 4 (*Glycine max*)73,458,878 (putative peroxygenase 4	PPER4	GCTTCCATCATAAACACTTCGG	62.13	AGGAAGGATTGGTGGCTTGGTT	64.61
Transcript ID: TRINITY_DN61766_c0_g1_i1					
Zinc finger CCH domain containing protein 69 –like isoform X1	ZF	GAGCCTGTCTGAAAGGGGAGCA	64.24	TGCAGCGACTACCATAAGCACA	62.3
Transcript ID: TRINITY_DN56363_c0_g1_i13					
AP2 like ethylene responsive transcription factor ANT like	AP2TF	ACTGTGGGGTGTGGAGAGTTGCA	66.09	GCCCTCTCTTCTTTGCATCCACAGC	66.04
Transcript ID:					
TRINITY_DN55029_c2_g4_i10					
Glyceraldehyde 3 phosphate dehyadrogenase 2,cytolistic	G3PDC	GCCCTCTGACTCCTCCTTGATAGCA	65.42	GGCATTCCGTGTCCCTACTGTGGA	66.28
Transcript ID: TRINITY_DN8658_c0_g1_i1					
Cellulose synthase A catalytic subunit	CS	AACTCACCAGACATCGGTTGCCC	65.11	AAGTCGGGGATGCTGTGGGAAGA	65.72
Transcript ID: TRINITY_DN51118_c0_g1_i8					
Auxin response Factor 3	ARF3	CCCATTTCATGTGACTCTTCTG	63.63	TGGATTCCAAAGAGCTGAACCT	61.5
Transcript ID: TRINITY_DN22465_c0_g1_i1					
Sucrose phosphate Synthase 3	SPS3	AGCCACATTGAGTCCCCAACGG	65.07	TGCGAGGCCTACGTTGTCATCCT	65.98
Transcript ID: TRINITY_DN21034_c0_g1_i1					
Ehtylene Respoonsive Trnscription Factor ERF012	ERTF	TCAGCAGAAACTCCACAAGCGA	62.43	TGGTATGACTTGGAGGGGTTGCA	63.77
Transcript ID: TRINITY_DN9541_c0_g1_i1					

### Data submission

The sequencing dataset used in the study is available in the NCBI repository with BioProject: PRJNA472133, BioSamples: SAMN09227257 (Asymptomatic flower bud tissue of soybean control) and SAMN09227258 (Symptomatic flower bud tissue of soybean infected with phytoplasma).

## Conclusions

This is the first transcriptomic study of FBD or WBS disease in soybean. We report transcriptomic signature of 17,454 DEGs involving 139 pathways in manifestation of the disease. The study reveals abnormal flower development at molecular level in terms of signalling, mobilization of carbohydrate and protein, phyllody, abnormal pollen development, improved colonization of insect in host plants. A total of 176,029 genic region putative markers (SSRs, SNPs and InDels) are reported which can be used as a genomic resource for future molecular breeding program for transfer of phytoplasma resistance in soybean variety improvement. Such more studies are warranted not only for understanding the disease at molecular level but they are also required for germplasm improvement in the endeavour of soybean productivity.

## Methods

### Identification of symptomatic and asymptomatic plant of FBD

For present investigation, the most popular widely grown soybean variety, JS- 335 of India was selected where recent FBD outbreak has adversely affected its productivity. Experimental data and tissue were collected from the experimental field of Department of Agricultural Botany, Dr. Panjabrao Deshmukh Krishi Vidyapeeth, Akola located in tropical belt at 307.4 m above mean sea level. The geographical situation is 20.42°N latitude and 77.02°E longitude with medium black, with clay, fairly levelled and uniform in topography with appropriate drainage. To discriminate symptomatic and asymptomatic plants, pollen viability, morphology by simple and scanning electron microscope (SEM), stigma receptivity assay, anther morphology and germination assay was done at R2 reproductive stage as previously described [12].

### PCR based test for diagnosis of phytoplasma

Symptomatic and asymptomatic plants were diagnosed by PCR based test from plant leaf tissue samples collected. DNA isolation was done using MO BIO PowerPlant Pro DNA Isolation Kit (Carlsbad, CA) as per manufacturer’s protocol. PCR reaction was carried out with 1X PCR buffer, 1.5 mM MgCl2, 50 ng genomic DNA, 200 μM of dNTPs, 1 unit of Taq enzyme (Invitrogen, Germany), 0.5% DMSO, 2 μg/μl BSA, 1 μM of P1 and P7 primers [49]. Subsequently nested PCR was performed using primer R16F2n and R16R2 [50] where 10 fold dilution of initial PCR product were used as template.

### Tissue collection and RNA extraction

Three sets of symptomatic and asymptomatic plant tissues (frozen bud and node tissues) at R5 stage (1/8 in. long seed in the pod at one of the four uppermost nodes on the main stem) were collected and treated with RNAlater and stored at -80 °C.

For RNA extraction, sample pooling of 10 biological replicates of symptomatic and asymptomatic plant tissues were done to minimize the across sample variability in each set [51]. PureLink_RNA Mini Kit (Invitrogen, San Diego, USA) was used as per manufacturer’s protocol.

Quality of RNA was checked on the Bioanalyzer 2100 (Agilent Technologies) using Agilent RNA chip. Samples with > = 8 RNA Integrity Number (RIN) value were used for further analysis. RNA-seq library was prepared by TruSeq RNA Sample Prep Kits (Illumina) using 4 microgram of total RNA. Poly-A containing mRNA molecules were separated by poly-T oligo-attached magnetic beads as per manufacturer’s protocol. Fragmentation of mRNA was done using divalent cations at elevated temperature. cDNA synthesis was done using reverse transcriptase and random primers and second strand cDNA synthesis was done using DNA Pol I and RNase H. By an end repair process single ‘A’ gets added to ligate the adapters. Final cDNA library was created after purification and enrichment. To assess mRNA quality, enrichment, fragment size, and library size Bioanalyzer was used. Before sequencing, quantity of library was measured using Qubit and qPCR. Constructed libraries of single set were sequenced on Illumina HiSeq 2000 platform and 60 million reads (6GB) data were generated (2 x100bp PE reads).

### Pre-processing and de novo transcriptome assembly

Paired-end reads of *Glycine max* (Soybean) were generated using Illumina HiSeq2000 technology. Raw reads were assessed and visualized using FastQC tool [52]. For trimming and removal of low quality reads (bases from 3′ and 5′ end; phred-score ≤ 20, trimmomatic tool version 0.38 [53] was used. De novo transcriptome assembly was done using Trinity [54] and redundant sequences were removed by CAP3 [55]. Being interactome data, having transcripts from two genomes, namely, soybean and phytoplasma, standard approach of aligning/filtering the reads on a reference genome was not followed. This was done in order to get “extra genes” along with isoforms and also to retain phytoplasma transcripts in subsequent analysis.

### Identification of differentially expressed genes

De novo transcriptome assembly was used for identification of DEGs. Mapping and alignment of raw reads over de novo transcriptome assembly was done using Bowtie [56]. RNA-Seq by Expectation-Maximization (RSEM) tool [57] was used to calculate expression values of each transcript in terms of Fragments per kilobase of exon per million mapped reads. Subsequently, for identification of differential expressed genes, edgeR (Empirical analysis of Digital Gene Expression in R) [58] was used which normalizes the data using TMM (Trimmed Mean of M-values) method [59]. To reduce the noise and computational reproducibility, NOIseq tool was used which is having both non-parametric and data-adaptive approach for the identification of differential expressed genes is based on count matrix [60]. For the expression analysis, threshold variance and *P*-value was set to 0.05 for identification of significant genes [61, 62]. A comparative approach for identification of DEG by both these tools were done at three different q values, i.e. q = 0.9, q = 0.95 and q = 0.99.

### Annotation and functional characterization

Differential expressed genes were taken for homology search against NCBI non-redundant database using Blastx algorithm (standalone local ncbi-blast-2.2.31+; E-value 1e-5) [63]. Blast2Go Pro version 3.1 software [64] was employed for annotation and functional characterization of the DEGs. Transcriptional factors prediction was done using PlantTFDB v4.0 database [65].

### Mining of putative molecular markers

Putative molecular markers, namely, simple sequence repeats (SSRs), single nucleotide polymorphism (SNPs) and InDels were mined from transcriptomic data. MISA-MIcroSAtellite identification tool [66] was used to mine the putative SSR markers using PERL script. Repeat units used were 10 and 6 for mono- and dinucleotides, respectively. For tri-, tetra-, penta- and hexa-nucleotides, 5 repeat units were used. This was followed by generation of primers of SSR loci using PRIMER3 tool [67]. Mined SSRs having di and tri repeats were used for in silico PCR validation. Transcripts with more than 1000 bp were selected and primers were designed for e-PCR based genotyping. e-PCR products having more than one hit in the entire soybean genome were excluded.

Since transcriptomic data are generated from single Indian genotype (JS- 335) variety thus reference genome of USA variety, Williams 82 can be used for SNP mining. In order to discover SNP using reference based mapping. SNP (Single Nucleotide Polymorphism) calling was performed against reference genome of *Glycine max* v2.0 using several filters like minimum read depth coverage > = 15 [68], Quality score > 30 [69, 70] and considering all the variants with minimum 50 bp flaking region from both sides. Reference soybean genome assembly (*Glycine max* v2.0 under accession number GCA_000004515.3) was retrieved from NCBI for mining of SNPs and InDels from soybean transcriptome using Burrows-Wheeler Aligner [71] and Samtools [72]. Further, annotations of identified variants were performed by using SnpEff tool [73].

### Validation and expression analysis by qPCR

cDNA synthesis was done for each RNA sample using RevertAid First Strand cDNA Synthesis Kit (Genetix, USA) to obtain template for qPCR. From DEG, 10 transcripts were randomly selected (5 up and 5 down-regulated) for designing of primer by Primer3 [67]. PCR was carried out in triplicate using QuantiFast SYBR Green PCR Master Mix kit (Genetix, USA) on real time PCR machine (ABI-7300, Applied Biosystem) for 40 cycles with melt curve step. For normalization, housekeeping gene, actin was used as reference. PCR optimization was done to obtain linear relationship for each set of primer pair. Finally, ΔΔCT fold change value [74] was calculated to obtain magnitude of differential gene expression. It was compared with logFC value for validation.

## Additional file

Additional file 1: Heatmap for the graphical representation of differential expressed genes found in Infected vs. control sample (DOCX 50 kb)

Additional file 2: Expression profile and blast results of differential expressed genes. (XLSX 2004 kb)

Additional file 3: Blast results of identified transcriptional factors found in DEGs. (XLSX 599 kb)

Additional file 4: Pathways identified in differential expressed genes of soybean. (XLSX 55 kb)

Additional file 5: List of identified SSR and three sets of designed three sets of primers. (XLSX 5310 kb)

Additional file 6: List of 193 SSR loci by e-PCR. (XLSX 24 kb)

Additional file 7: Variants identified from soybean samples against reference genome of *Glycine max* and also annotation of variants. (XLSX 31287 kb)

## Abbreviations

cDNA: complementary DNA
DEG: Differential Expressed Genes
DNA: Deoxyribonucleic Acid
edgeR: Empirical analysis of Digital Gene Expression in R
FBD: Floral Bud Distortion
InDel: Insertion-Deletion
LYD: Lethal Yellowing Disease
NCBI: National Center for Biotechnology Information
PCR: Polymerase Chain Reaction
PERL: Practical Extraction and Report Language
PlantTFDB: Plant Transcription Factor Database
RIN: RNA Integrity Number
RNA: Ribonucleic Acid
RSEM: RNA-Seq by Expectation-Maximization
SEM: Scanning Electron Microscope
SNP: Single Nucleotide Polymorphism
SSR: Simple Sequence Repeats
TF: Transcription Factor
WBS: Witches’ Broom Syndrome

## References

[1] Horvath AA (1926). Changes in the blood composition of rabbits fed on raw soy beans. J Biol Chem.

[2] Masuda T, Goldsmith PD (2009). World soybean production: area harvested, yield, and long-term projections. International food and agribusiness management review.

[3] Friedman M, Brandon DL (2001). Nutritional and health benefits of soy proteins. J Agric Food Chem.

[4] Popp J, Harangi-Rákos M, Gabnai Z, Balogh P, Antal G, Bai A (2016). Biofuels and their co-products as livestock feed: global economic and environmental implications. Molecules.

[5] Miransari M, editor. Abiotic and biotic stresses in soybean production: soybean production. Academic press; 2015.

[6] Jadhav Pravin V, Mane SS, Nandanwar RS, Kale PB, Dudhare MS, Moharil MP (2013). Floral bud distortion in soybean and incidence in Central India. Egypt J Biol.

[7] Subekti NA (2008). The effects of disease on plant reproduction as basis for breeding for disease resistance. In Proceedings of the scientific seminar and annual meeting of the regional commissioner PEI PFI XIX South Sulawesi.

[8] Marr DL, Marshall ML (2006). The role of fungal pathogens in flower size and seed mass variation in three species of Hydrophyllum (Hydrophyllaceae). Am J Bot.

[9] Pracros P, Renaudin J, Eveillard S, Mouras A, Hernould M (2006). Tomato flower abnormalities induced by stolbur phytoplasma infection are associated with changes of expression of floral development genes. Mol Plant-Microbe Interact.

[10] Singh AK, Bhatt BP (2013). Occurrence of phytoplasma phyllody and witches' broom disease of faba bean in Bihar. J Environ Biol.

[11] Sugano J, Melzer M, Pant A, Radovich T, Fukuda S, Migita S, et al. Field evaluations of tomato yellow leaf curl virus-resistant varieties for commercial production. Plant Dis. 2011.

[12] Kale PB, Jadhav PV, Wakekar RS, Moharil MP, Deshmukh AG, Dudhare MS (2016). Cytological behaviour of floral organs and in silico characterization of differentially expressed transcript-derived fragments associated with ‘floral bud distortion’in soybean. J Genet.

[13] Dhingra KL, Chenulu VV (1983). Symptomatology and transmission of witches' broom disease of soybean in India. Current science (India).

[14] Thorat V, More V, Jadhav P, Mane SS, Nandanwar RS, Surayavanshi M (2016). First report of a 16SrII-D group Phytoplasma associated with witches’-broom disease of soybean (Glycine max) in Maharashtra, India. Plant Dis.

[15] Kumar S, Sharma P, Sharma S, Rao GP (2015). Mixed infection and natural spread of ‘Candidatus Phytoplasma asteris’ and Mungbean yellow mosaic India virus affecting soya bean crop in India. J Phytopathol.

[16] K. Subramanya Sastry. Seed-borne plant virus diseases. Springer India 2013, Springer Science & Business Media. ISBN 978-81-322-0812-9. http://www.soybeancheckoffresearch.org/DetailsbyPaperid.php?id_Paper=1144. Accessed August 2014.

[17] Golnaraghi AR, Shahraeen N, Pourrahim R, Farzadfar S, Ghasemi A (2004). Occurrence and relative incidence of viruses infecting soybeans in Iran. Plant Dis.

[18] Rahimian H, Hamdollah-Zadeh A, Montazeri M. Viruses associated with the soybean pod set failure syndrome in Iran. In Proceedings of the 12th Iranian Plant Protection Congress 2–7 September 1995. Karadj (Iran Islamic Republic). 1995.

[19] Wong CE, Singh MB, Bhalla PL (2009). Molecular processes underlying the floral transition in the soybean shoot apical meristem. Plant J.

[20] Severin AJ, Woody JL, Bolon YT, Joseph B, Diers BW, Farmer AD (2010). RNA-Seq atlas of Glycine max: a guide to the soybean transcriptome. BMC Plant Biol.

[21] Jung CH, Wong CE, Singh MB, Bhalla PL (2012). Comparative genomic analysis of soybean flowering genes. PLoS One.

[22] Schmutz J, Cannon SB, Schlueter J, Ma J, Mitros T, Nelson W (2010). Genome sequence of the palaeopolyploid soybean. Nature.

[23] Liu SC, Jin JQ, Ma JQ, Yao MZ, Ma CL, Li CF (2016). Transcriptomic analysis of tea plant responding to drought stress and recovery. PLoS One.

[24] Iovieno P, Punzo P, Guida G, Mistretta C, Van Oosten MJ, Nurcato R (2016). Transcriptomic changes drive physiological responses to progressive drought stress and rehydration in tomato. Front Plant Sci.

[25] Fu L, Ding Z, Han B, Hu W, Li Y, Zhang J (2016). Physiological investigation and transcriptome analysis of polyethylene glycol (PEG)-induced dehydration stress in cassava. Int J Mol Sci.

[26] Shin JH, Vaughn JN, Abdel-Haleem H, Chavarro C, Abernathy B, Do Kim K (2015). Transcriptomic changes due to water deficit define a general soybean response and accession-specific pathways for drought avoidance. BMC Plant Biol.

[27] Bove JM (1981). Mycoplasma infections of plants. Isr J Med Sci.

[28] Panchy N, Lehti-Shiu M, Shiu SH (2016). Evolution of gene duplication in plants. Plant Physiol.

[29] Yang S, Tang F, Zhu H (2014). Alternative splicing in plant immunity. Int J Mol Sci.

[30] Kazemian M, Ren M, Lin JX, Liao W, Spolski R, Leonard WJ (2015). Comprehensive assembly of novel transcripts from unmapped human RNA-Seq data and their association with cancer. Mol Syst Biol.

[31] Palmieri N, Nolte V, Suvorov A, Kosiol C, Schlötterer C (2012). Evaluation of different reference based annotation strategies using RNA-Seq–a case study in Drososphila pseudoobscura. PLoS One.

[32] Bai X, Zhang J, Ewing A, Miller SA, Radek AJ, Shevchenko DV (2006). Living with genome instability: the adaptation of phytoplasmas to diverse environments of their insect and plant hosts. J Bacteriol.

[33] Marcone C, Neimark H, Ragozzino A, Lauer U, Seemüller E (1999). Chromosome sizes of phytoplasmas composing major phylogenetic groups and subgroups. Phytopathology.

[34] Mou HQ, Lu J, Zhu SF, Lin CL, Tian GZ, Xu X (2013). Transcriptomic analysis of paulownia infected by paulownia witches'-broom phytoplasma. PLoS One.

[35] Lu YT, Li MY, Cheng KT, Tan CM, Su LW, Lin WY (2014). Transgenic plants that express the phytoplasma effector SAP11 show altered phosphate starvation and defense responses. Plant Physiol.

[36] Kube M, Mitrovic J, Duduk B, Rabus R, Seemüller E. Current view on phytoplasma genomes and encoded metabolism. Sci World J. 2012;2012. 10.1100/2012/185942 PMC3322544 22550465

[37] Andersen MT, Liefting LW, Havukkala I, Beever RE (2013). Comparison of the complete genome sequence of two closely related isolates of ‘Candidatus Phytoplasma australiense’reveals genome plasticity. BMC Genomics.

[38] Seemüller E, Sule S, Kube M, Jelkmann W, Schneider B (2013). The AAA+ ATPases and HflB/FtsH proteases of ‘Candidatus Phytoplasma Mali’: phylogenetic diversity, membrane topology, and relationship to strain virulence. Mol Plant-Microbe Interact.

[39] Orlovskis Z. Role of phytoplasma effector proteins in plant development and plant-insect interactions (doctoral dissertation, University of East Anglia). 2017.

[40] Yang J, Tian L, Sun MX, Huang XY, Zhu J, Guan YF (2013). AUXIN RESPONSE FACTOR17 is essential for pollen wall pattern formation in Arabidopsis. Plant Physiol.

[41] Wakekar RS, Jadhav PV, Kale PB, Moharil MP, Nandanwar RS, Mane SS (2018). Pollen dysfunction causes ‘floral bud Distortion’in Indian soybean (Glycine max). Agricultural Research.

[42] Kaminska M, Berniak H, Kaminski P (2012). Failure of flower bud formation in Brassica plants associated with phytoplasma infection. J Agric Sci.

[43] Sugio A, MacLean AM, Grieve VM, Hogenhout SA (2011). Phytoplasma protein effector SAP11 enhances insect vector reproduction by manipulating plant development and defense hormone biosynthesis. Proc Natl Acad Sci.

[44] Sugio A, MacLean AM, Kingdom HN, Grieve VM, Manimekalai R, Hogenhout SA (2011). Diverse targets of phytoplasma effectors: from plant development to defense against insects. Annu Rev Phytopathol.

[45] Anthouard R, DiRita VJ (2015). Chemical biology applied to the study of bacterial pathogens. Infect Immun.

[46] Lenz RR, Dai W. Mapping X-disease Phytoplasma resistance in Prunus virginiana. Front Plant Sci. 2017;8. 10.3389/fpls.2017.02057 PMC5712551 29238359

[47] Jarausch W, Bisognin C, Schneider B, Grando MS, Velasco R, Seemüller E (2007). Breeding of apple rootstocks resistant to Candidatus phytoplasma Mali. Bulletin of Insectology.

[48] Gurr GM, Johnson AC, Ash GJ, Wilson BA, Ero MM, Pilotti CA (2016). Coconut lethal yellowing diseases: a phytoplasma threat to palms of global economic and social significance. Front Plant Sci.

[49] Smart CD, Schneider B, Blomquist CL, Guerra LJ, Harrison NA, Ahrens U (1996). Phytoplasma-specific PCR primers based on sequences of the 16S-23S rRNA spacer region. Appl Environ Microbiol.

[50] Gundersen DE, Lee IM, Schaff DA, Harrison NA, Chang CJ, Davis RE (1996). Genomic diversity and differentiation among phytoplasma strains in 16S rRNA groups I (aster yellows and related phytoplasmas) and III (X-disease and related phytoplasmas). Int J Syst Evol Microbiol.

[51] Zou C, Wang P, Xu Y (2016). Bulked sample analysis in genetics, genomics and crop improvement. Plant Biotechnol J.

[52] Andrew S (2010). FastQC: a quality control tool for high throughput sequence data.

[53] Bolger AM, Lohse M, Usadel B (2014). Trimmomatic: a flexible trimmer for Illumina sequence data. Bioinformatics.

[54] Haas BJ, Papanicolaou A, Yassour M, Grabherr M, Blood PD, Bowden J (2013). De novo transcript sequence reconstruction from RNA-seq using the trinity platform for reference generation and analysis. Nat Protoc.

[55] Huang X, Madan A (1999). CAP3: A DNA sequence assembly program. Genome Res.

[56] Langmead B, Trapnell C, Pop M, Salzberg SL (2009). Ultrafast and memory-efficient alignment of short DNA sequences to the human genome. Genome Biol.

[57] Li B, Dewey CN (2011). RSEM: accurate transcript quantification from RNA-Seq data with or without a reference genome. BMC bioinformatics..

[58] Robinson MD, McCarthy DJ, Smyth GK (2010). edgeR: a Bioconductor package for differential expression analysis of digital gene expression data. Bioinformatics.

[59] Robinson MD, Oshlack A (2010). A scaling normalization method for differential expression analysis of RNA-seq data. Genome Biol.

[60] Tarazona S, García-Alcalde F, Dopazo J, Ferrer A, Conesa A (2011). Differential expression in RNA-seq: a matter of depth. Genome Res.

[61] Sotak M, Czeranková O, Klein D, Jurčacková Z, Li L, Čellárová E (2016). Comparative transcriptome reconstruction of four Hypericum species focused on Hypericin biosynthesis. Front Plant Sci.

[62] Wang Y, Guo ZY, Sun X, Lu SB, Xu WJ, Zhao Q (2016). Identification of changes in gene expression of rats after sensory and motor nerves injury. Sci Rep.

[63] Camacho C, Coulouris G, Avagyan V, Ma N, Papadopoulos J, Bealer K (2009). BLAST+: architecture and applications. BMC bioinformatics.

[64] Conesa A, Götz S, García-Gómez JM, Terol J, Talón M, Robles M (2005). Blast2GO: a universal tool for annotation, visualization and analysis in functional genomics research. Bioinformatics.

[65] Jin J, Tian F, Yang DC, Meng YQ, Kong L, Luo J, et al. PlantTFDB 4.0: toward a central hub for transcription factors and regulatory interactions in plants. Nucleic Acids Res 2016:gkw982. 10.1093/nar/gkw982 PMC5210657 27924042

[66] Thiel T, Michalek W, Varshney R, Graner A (2003). Exploiting EST databases for the development and characterization of gene-derived SSR-markers in barley (Hordeum vulgare L.). Theor Appl Genet.

[67] Untergasser A, Cutcutache I, Koressaar T, Ye J, Faircloth BC, Remm M (2012). Primer3—new capabilities and interfaces. Nucleic Acids Res.

[68] Uitdewilligen JG, Wolters AM, Bjorn B, Borm TJ, Visser RG, van Eck HJ (2013). A next-generation sequencing method for genotyping-by-sequencing of highly heterozygous autotetraploid potato. PLoS One.

[69] Liu J, McCleland M, Stawiski EW, Gnad F, Mayba O, Haverty PM (2014). Integrated exome and transcriptome sequencing reveals ZAK isoform usage in gastric cancer. Nat Commun.

[70] Yu X, Sun S (2013). Comparing a few SNP calling algorithms using low-coverage sequencing data. BMC bioinformatics..

[71] Li H, Durbin R (2009). Fast and accurate short read alignment with burrows–wheeler transform. Bioinformatics.

[72] Li H, Handsaker B, Wysoker A, Fennell T, Ruan J, Homer N (2009). The sequence alignment/map format and SAMtools. Bioinformatics.

[73] Cingolani P, Platts A, Wang LL, Coon M, Nguyen T, Wang L (2012). A program for annotating and predicting the effects of single nucleotide polymorphisms, SnpEff: SNPs in the genome of Drosophila melanogaster strain w1118; iso-2; iso-3. Fly (Austin).

[74] Livak KJ, Schmittgen TD (2001). Analysis of relative gene expression data using real-time quantitative PCR and the 2− ΔΔCT method. Methods.

