# Supplementary material for: Transcriptomic signature reveals mechanism of flower bud distortion in witches’-broom disease of soybean (Glycine max)
Source: BMC Plant Biol. 2019 Jan 15;19:26. doi: 10.1186/s12870-018-1601-1 (PMC6332543; doi:10.1186/s12870-018-1601-1)

**Additional file 1:** Heatmap for the graphical representation of differential expressed genes found in Infected vs. control sample


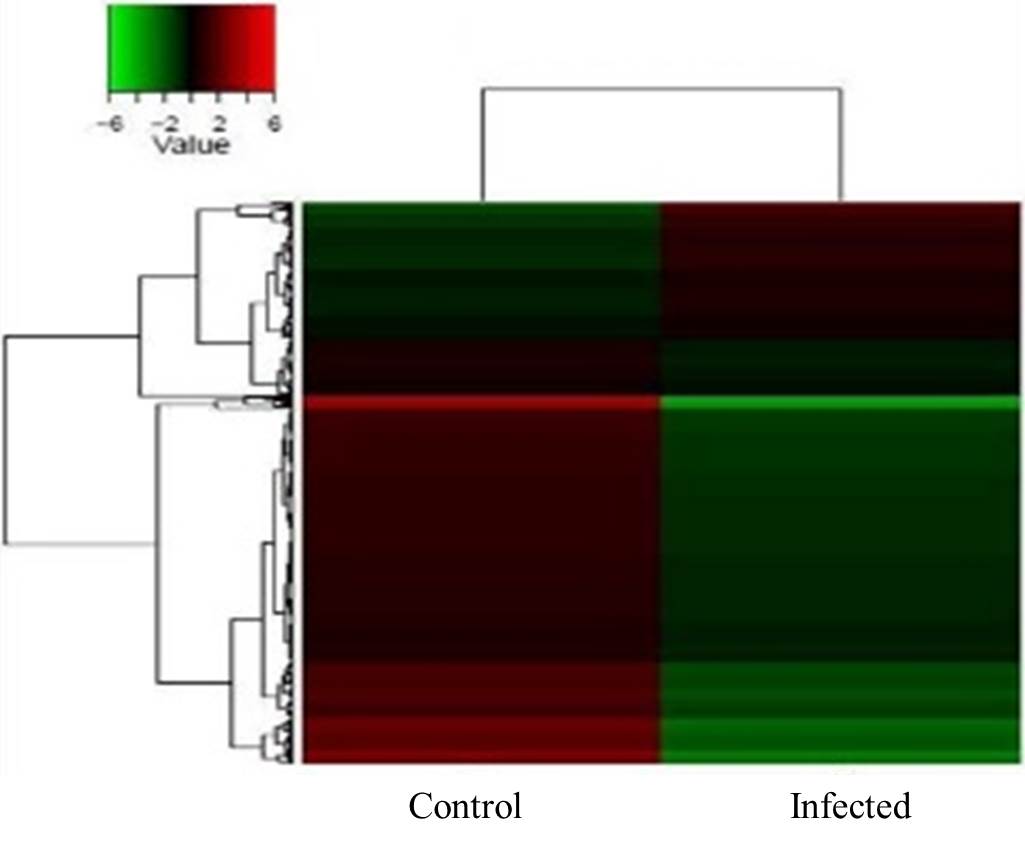

Supplement: Supplementary file 1 — Heatmap for the graphical representation of differential expressed genes found in Infected vs. control sample (DOCX 50 kb) [file 12870_2018_1601_MOESM1_ESM.docx]
